# Supplementary material for: Preparation and Characterization of Nano-Fe3O4 and Its Application for C18-Functionalized Magnetic Nanomaterials Used as Chromatographic Packing Materials
Source: Nanomaterials (Basel). 2023 Mar 20;13(6):1111. doi: 10.3390/nano13061111 (PMC10058610; doi:10.3390/nano13061111)
Supplement: Supplementary file 1 [file nanomaterials-13-01111-s001.zip › nanomaterials-2274148-supplementary.pdf]

Supplementary Materials

Preparation and Characterization of Nano-Fe_3O_4 and Its Application for C18-Functionalized Magnetic Nanomaterials Used as Chromatographic Packing Materials

Wen-Xin Liu [1,2], Wei-Na Zhou [2], Shuang Song [1], Yong-Gang Zhao [2,*] and Yin Lu [2,*]

[1] College of Environment, Zhejiang University of Technology, Hangzhou 310014, China; lwx187221@163.com (W.-X.L.); ss@zjut.edu.cn (S.S.)
[2] College of Biological and Environmental Engineering, Zhejiang Shuren University, Hangzhou 310015, China; 18030219706@163.com
* Correspondence: zhyg91213@163.com (Y.-G.Z.); luyin@zjsru.edu.cn (Y.L.)

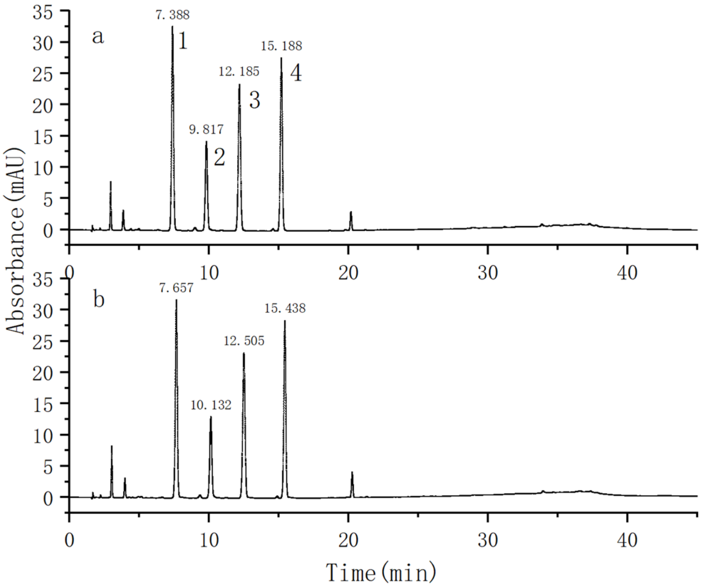

Figure S1. Chromatograms of the four sulfonamides: (1) sulfamethyldiazine; (2) sulfamethazine; (3) sulfamethoxypyridazine; and (4) sulfamethoxazole. (**a**) Diamonsil C18 (5 µm, 4.6 × 250 mm); (**b**) Self-made chromatographic column (5 µm, 4.6 × 250 mm).

Table S1. Chemical structure of reagents used for the preparation of C18@PS-DVB-Fe_3O_4 particles.

Number	Reagents	Chemical Structures
1	Styrene (ST)	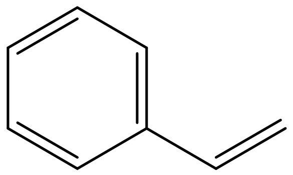
2	Oleic acid (OA)	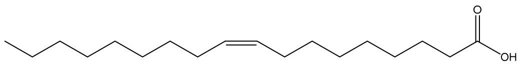
3	Divinylbenzene (DVB)	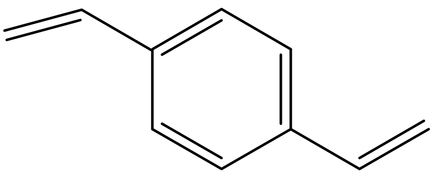

4	2,2′-Azobis(2-methylpropionitrile) (AIBN)	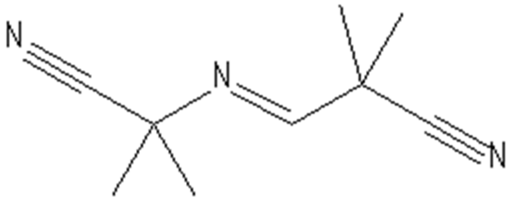
5	Sodium dodecyl sulfate (SDS)	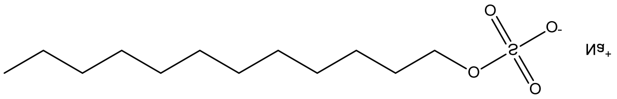
6	Dibutyl phthalate (DBP)	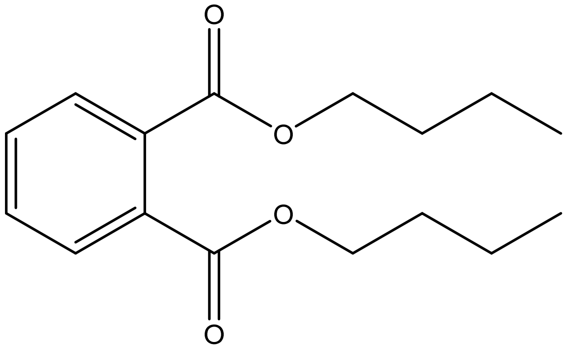
7	Benzoyl peroxide (BPO)	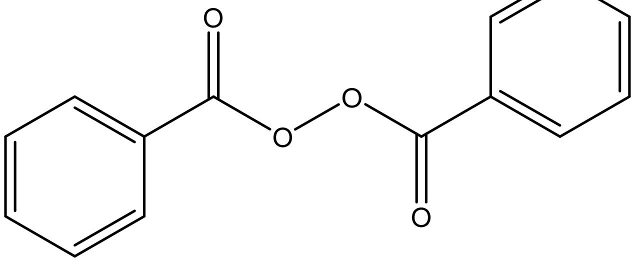
8	N, N-dimethyloctadecylamine	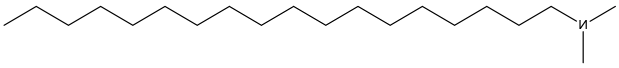
9	Glycidyl methacrylate (GMA)	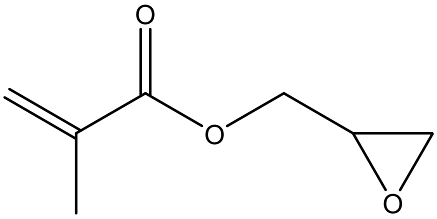
